# P-397. Improving Viral Suppression Among Out-of-Care Older People With HIV in a Humanitarian Context via Peer-Run Optimal Strategy for Treatment (PROST) in Ukraine: Pilot Study Results

**DOI:** 10.1093/ofid/ofaf695.614

**Published:** 2026-01-11

**Authors:** Oleksandr Zeziulin, Iryna Zaviriukha, Kostyantyn Dumchev, Iryna Kharandiuk, Vladyslav Fedorchenko, Alexandra A Deac, Sheela Shenoi, Julia Rozanova

**Affiliations:** Ukrainian Institute for Public Health Policy, Kyiv, Kyyiv, Ukraine; European Institute of Public Health Policy, Kyiv, Kyyiv, Ukraine; UIPHP, Kyiv, Kyyiv, Ukraine; European Institute of Public Health Policy, Kyiv, Kyyiv, Ukraine; Independent Researcher, Kyiv, Kyyiv, Ukraine; King’s College London & University of East London, London, England, United Kingdom; Yale University, New Haven, CT; Yale School of Medicine, New Haven, Connecticut

## Abstract

**Background:**

Ukraine has the highest HIV prevalence in Europe at 1.2%. Antiretroviral therapy (ART) coverage is significantly lower among older people living with HIV(OPWH)(50+). OPWH have more challenges in treatment versus younger individuals. They often receive late diagnoses and require management of opportunistic infections and other chronic conditions. Russia's full-scale invasion in 2022 disrupted PWH by affecting the economy and healthcare services that lost 35,000 clinicians since the invasion.

**Methods:**

Peer support helps re-engage and retain OPWH in HIV care, especially during crises. The Peer-Run Optimal Support for Treatment (PROST) intervention was developed using the ADAPT-ITT framework. This process involved a multidisciplinary team, including OPWH, HIV clinicians, clinical psychologists, and social workers who work with OPWH. From February 2023 to April 2024, we tested the PROST intervention at Kyiv's largest HIV clinic to evaluate its feasibility, acceptability, and preliminary effectiveness among out-of-care clients (OOC) with no-ART-refill for over 90 days and ART-naïve OPWH. Enrolled participants were randomized 2:1 into PROST or treatment as usual (TAU) group. Both arms were surveyed, and HIV clinical data were extracted at baseline, 3 and 6-month follow-up.

**Results:**

Study enrolled 56 OOC and 37 newly diagnosed ART-naïve OPWH. The mean age was 56.4 years (SD=5.6) and 55 % were women; randomization worked with no significant difference between the PROST vs TAU arm on any characteristic. Study retention rates at three and six months were 93.5% and 91.4%, respectively, with similar rates in both the PROST and TAU arms. At month three, the proportion of OOC achieving viral suppression (VS)(< 200 copies) was significantly higher in the PROST arm compared to TAU (63.4% vs. 53.3%; p< 0.001). No significant benefits were observed in the ART-naïve group. For OOC at month six, being in the PROST arm and experiencing reduced depression symptoms was associated with VS (OR=0.15, 95% CI: 0.02–0.94, p=0.042).

**Conclusion:**

PROST intervention may decrease depressive symptoms and help to achieve VS, including undetectable viral loads among OOC OPWH. A fully powered trial is necessary to confirm whether PROST can mitigate the negative effects of poor mental health on VS in this population.

**Disclosures:**

Sheela Shenoi, MD MPH, Merck Pharmaceuticals: My spouse worked for Merck 1997-2007 and retains company stock in his retirement account. There is no conflict of interest with this work.

